# Pendelluft as a predictor of weaning in critically ill patients: An observational cohort study

**DOI:** 10.3389/fphys.2023.1113379

**Published:** 2023-03-31

**Authors:** Danqiong Wang, Yaxin Ning, Linya He, Keqi Pan, Xiaohua Xiong, Shanshan Jing, Jianhua Hu, Jian Luo, Dehua Ye, Zubing Mei, Weiwen Zhang

**Affiliations:** ^1^ Department of Critical Care Medicine, The Quzhou Affiliated Hospital of Wenzhou Medical University, Quzhou People’s Hospital, Quzhou, China; ^2^ The Second School of Clinical Medicine, Zhejiang Chinese Medical University, Hangzhou, China; ^3^ School of Medicine, Shaoxing University, Shaoxing, China; ^4^ Institute of Teacher Education, Department of Mathematics and Physics, Quzhou University, Quzhou, China; ^5^ Department of Anorectal Surgery, Shuguang Hospital Affiliated to Shanghai University of Traditional Chinese Medicine, Shanghai, China; ^6^ Anorectal Disease Institute of Shuguang Hospital, Shanghai, China

**Keywords:** pendelluft, electrical impedance tomography, weaning, predict, mechanical ventilation

## Abstract

**Objective:** Weaning failure is associated with adverse clinical outcomes. This study aimed to evaluate the accuracy of pendelluft during the spontaneous breathing trials (SBT) as a predictor of weaning outcome of patients with mechanical ventilation.

**Methods:** An observational cohort study included 60 critically ill patients who were eligible for extubation. Pendelluft and electrical activity of the diaphragm (Edi) were monitored at baseline and every 10 minutes for the first 30 min of SBT denoted as T0, T1, T2, and T3. The pendelluft was measured using electrical impedance tomography (EIT), and Edi parameters were collected by Edi catheter. Patients were followed up after extubation and were divided into success group and failure group. Pendelluft, Edi parameters, respiratory parameters, and clinical outcomes such as intensive care units (ICU) stay, mortality, and 28-day ventilator-free days were compared between the two groups. Receiver operating characteristic (ROC) curves were constructed to evaluate the ability of pendelluft to predict weaning outcome.

**Results:** Fifty patients (50/60) were successfully weaned from the machine and 10 (10/60) failed, with weaning failure rate of 16.7%. Respiratory parameters such as rapid shallow breathing index (RSBI), respiratory rate (RR) and Edi parameters such as maximum value of Edi (Edimax), Edi variation between a maximum and minimum(ΔEdi) in the failure group were higher than those in the success group. The ICU stay and the 28-day ventilator-free days in the failure group were significantly longer than those in the success group. The 28-day mortality rate was higher in the failure group. The pendelluft mainly occurred in the early stage of SBT. Ventral pendelluft and total pendelluft in the failure group were higher than those in the success group at T1. Edimax and ΔEdi were positively correlated with pendelluft. The area under ROC curve (AUC) showed moderate predictive ability for ventral pendelluft in predicting weaning failure at T1 (AUC 0.76, 95% CI 0.58–0.94, cut-off value > 3% global tidal variation).

**Conclusion:** Pendelluft is one of the factors leading to weaning failure, which may be related to diaphragm function. Measuring pendelluft volume maybe helpful to predict weaning.

## 1 Introduction

As a life-saving technology, mechanical ventilation (MV) plays a vital role in the management of patients in intensive care unit (ICU). Studies have shown that the complications of mechanical ventilation will lead to increased treatment costs and adverse clinical outcomes (Schonhofer et al., 2020; He et al., 2021). Therefore, ventilator discontinuation should be considered as soon as the cause of respiratory failure is improved. However, despite the fact that various evaluation systems are available for clinical practice (da Silva Neto et al., 2020; Vu et al., 2020; Sato et al., 2021), the rate of weaning failure remains as high as 20%–54% (Burns et al., 2021; Zhao et al., 2021).

The commonly used evaluation indicators in clinical practice include P0.1 (Sato et al., 2021), rapid shallow breathing index (RSBI) (Souza and Lugon, 2015), inspiratory negative pressure (INF) (Vu et al., 2020), and ultrasound (Qian et al., 2018; Sharma et al., 2018). But in fact, it is difficult to continuously monitor the ventilation distribution of patients with these indicators. Improving alveolar collapse and hyperinflation is the key point for the treatment for these critically ill patients with respiratory failure, which is a prerequisite for weaning. As an individualized, non-invasive, real-time bedside imaging technology, electrical impedance tomography (EIT) (Frerichs et al., 2017) can be used as a potential tool for individualized management at different stages of ARDS (Jimenez et al., 2022) and monitor the regional ventilation distribution during weaning (Bickenbach et al., 2017). As we know, the commonly used treatment strategy is from controlled ventilation to assisted ventilation during weaning. However, inappropriate spontaneous breathing can lead to pendelluft (Yoshida et al., 2016). The phenomenon of pendelluft was found in 20.7% of patients receiving controlled ventilation and 40% of patients with spontaneous respiration (Chi et al., 2022). Patients who failed in SBT often experienced high pendelluft (Coppadoro et al., 2020), and pendelluft may be related to the insufficiency of diaphragm function (Hsu et al., 2017). However, whether the pendelluft is related to the weaning outcome remains unclear.

We, therefore, conducted this observational study to evaluate the pendelluft during SBT, in order to find potential useful parameters to predict weaning outcome.

## 2 Methods

This is a prospective observational study approved by the local ethics committee with the registration number of Shi 2018–006. Participation was entirely voluntary, and informed consent was obtained. This study was conducted in a medical ICU of a tertiary teaching hospital between January 2019 and December 2021.

### 2.1 Patients

ICU patients aged ≥ 18 years old who received mechanical ventilation for at least 24 h for the first SBT were consecutively included in this study. Inclusion criteria for this study were the following: (1) those who survived the acute disease phase that led to intubation;(2)PaO_2_/FiO2≥150 mmHg,PEEP ≤8 cmH_2_O,pH≥7.30,FiO_2_ <0.40;SO_2_≥90% (>85% in the presence of chronic respiratory failure); (3) those with stable hemodynamics with no need for vasopressors; (4) adequate ability to cough or Glasgow Coma Scale (GCS)≥13; (5) those with maintenance of water electrolyte balance.

Exclusion criteria included: (1) subjects with pregnancy or advanced cancer; (2) those with severe depression of central respiratory drive; (3) those with esophageal obstruction, esophageal perforation, esophageal variceal hemorrhage, or upper gastrointestinal surgery; (4) those with thoracic malformation or diaphragmatic hernia; (5) those with severe heart, liver, kidney and other organ failure, or hemodynamic instability; (6)those with severe coagulopathy; (7) those with broken skin on the chest affecting the placement of the EIT electrode; (8) patients with incomplete clinical data.

### 2.2 Study design and measurements

The patients were given controlled ventilation under baseline settings with a tidal volume of 8–10 mL/kg of predicting body weight, RR 12/min and PEEP 5 cm H_2_O. When patients met the indications for weaning, SBT was carried out by setting low-level pressure support ventilation (PSV). The support pressure was set at 6–8 cm H_2_O, and PEEP at 3–5 cm H_2_O for 30 min.

SBT failure was diagnosed clinically according to the following criteria: those with a blood oxygen saturation <90%; heart rate >120 beats/min or variability ≥20%; systolic blood pressure (SBP) > 180 mmHg or <90 mmHg or variability ≥20%; respiratory rate ≥35 breaths/min or variability ≥50%; or those with clinical signs of distress. In the absence of the above conditions, SBT was considered successful and oxygen therapy after extubation would be initiated.

An EIT belt was placed between the fifth or sixth ribs of the patient’s chest, which was connected to the EIT monitor to collect the pendelluft parameters. The Edi parameters were obtained through the Edi catheter, which was connected with the ventilator Edi signal monitoring module (Maquet Company, Sweden) for Edi data collection, including Edimax, Edimin, and ΔEdi (Edimax-Edimin). The parameters of pendelluft and Edi were recorded every 10 min for a total of 40 min, including the 10-min baseline and SBT_10 min, SBT_20 min, SBT_30 min, which were respectively represented by T0, T1, T2 and T3. Blood gas analysis was performed at baseline and after SBT.

Demographic data and short-term outcomes such as 28-day ventilator-free days (the number of days without invasive ventilation to day 28 of hospitalization), length of stay in ICU and 28-day mortality were collected.

### 2.3 Pendelluft volume measurement

**FIGURE 1:**
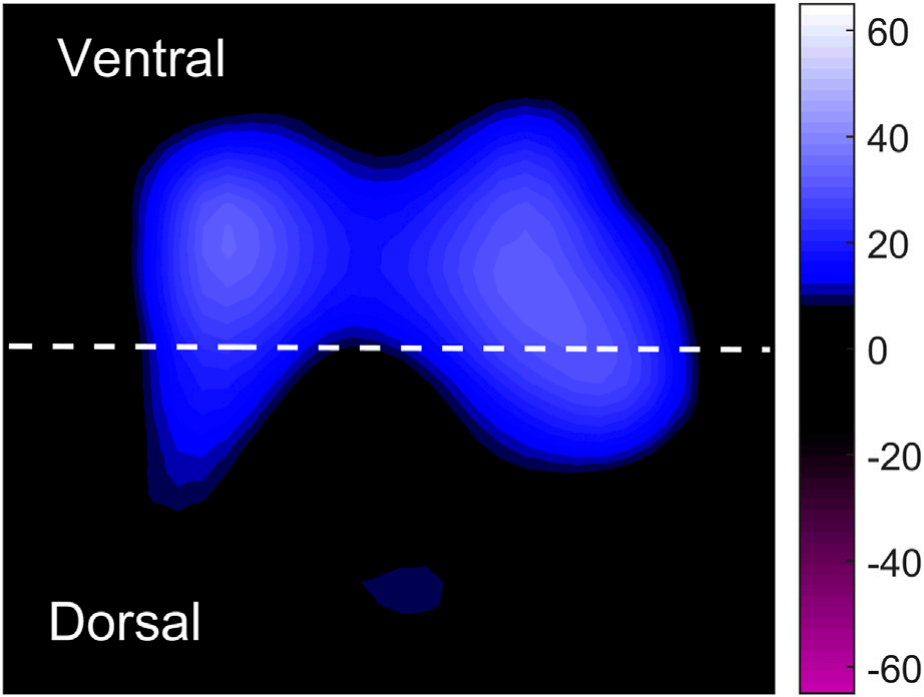
Schematic diagram for the tidal impedance variation of the entire lung. The whole lung was divided into ventral and dorsal equally, with dotted lines as the dividing line. Low ventilation area and high ventilation area are marked with dark blue and light blue, respectively. Tidal impedance variations at different phases are marked in purple.

**FIGURE 2:**
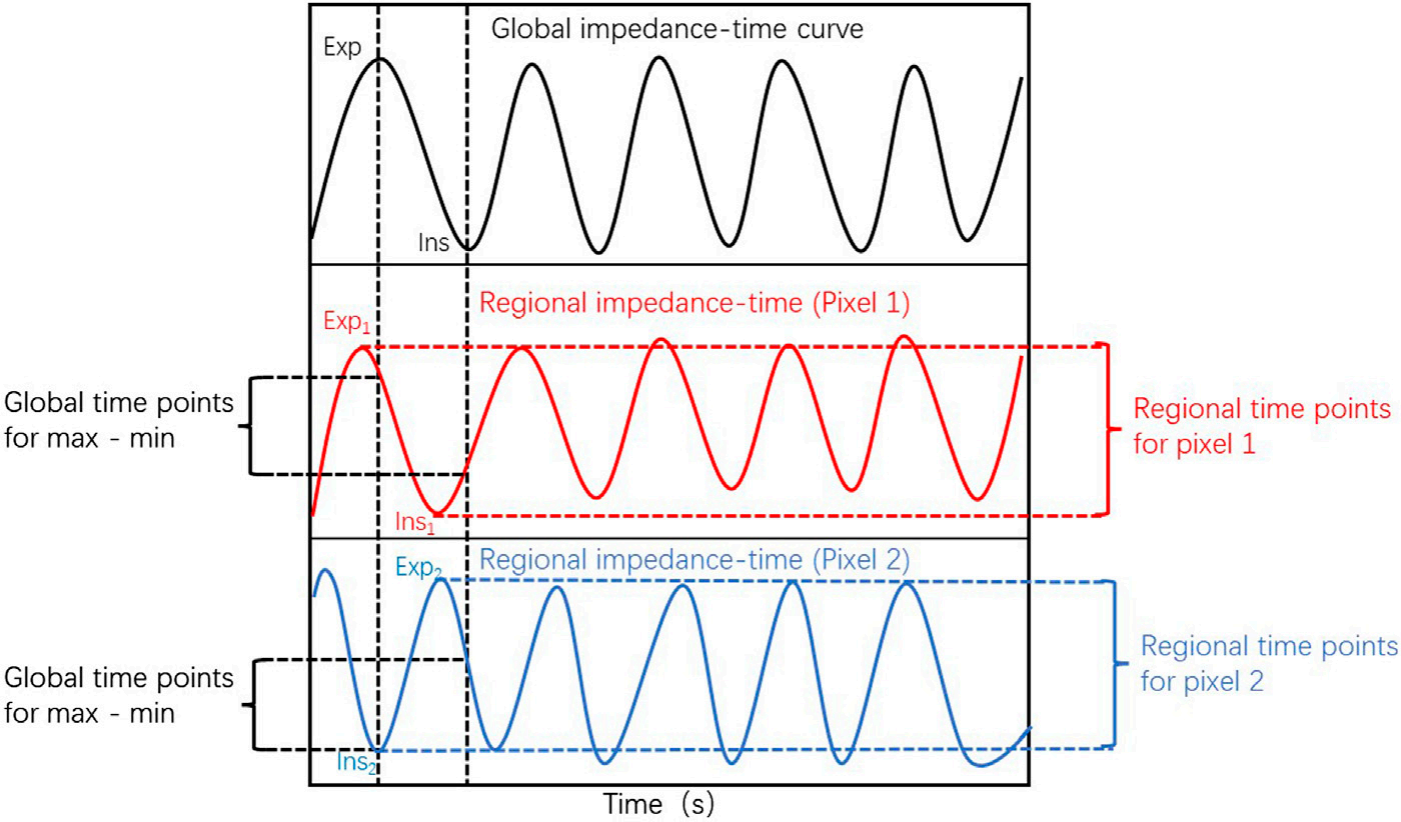
Pendelluft amplitude estimation based on electrical impedance tomography.The global impedance–time curve is presented in the upper row. The middle and bottom rows present the impedance–time curve from two representative pixels. The timepoints of nadir impedance (Ins) and peak impedance (Exp) are recognized as the starting and ending points of inspiration for global and regional pixels. The global tidal variation was defined as impedance difference between global Ins and Exp. The regional tidal variation was defined as impedance difference between regional Ins and Exp. The pendelluft volume was calculated as the difference between regional and global tidal variation.

EIT data were recorded with a sample frequency of 20 Hz and stored for offline analysis. As is reported by Sang et al. (2020) Pendelluft, defined as asynchronous alveolar ventilation, is caused by different regional time constants or dynamic pleural pressure variations, which can be evaluated by amplitude differences (defined as the impedance difference between sum of all regional tidal variation and the global tidal variation [GTV]), and it can be expressed as percentage of GTV as unit. The total pendelluft volume is the sum of the ventral and dorsal regions, and can also be understood as the total volume of pendelluft in the whole lung. EIT imaging was divided into two regions of interest including dorsal and ventral regions, which was presented in Figure 1. The region of interests for EIT were evenly divided into two regions, the upper and lower regions, which were defined as ventral region and dorsal region, respectively. The pendelluft volume in each region was analyzed. Pendelluft amplitude estimation based on EIT was shown in Figure 2. Data analyses were performed offline with MATLAB7.2 (The MathWorks Inc., Natick, United States).

The patients were divided into success group and failure group according to whether weaning was successful or not. Failure group was defined as follows: (1) those with SBT failure; (2) those requiring reintubation and/or the resumption of MV within 48 h of extubation; (3) those died within 48 h of extubation.

The criteria for reintubation were: patients with severe respiratory failure (at least two of the following criteria: respiratory rate of more than 35 times/min, clinical signs of respiratory distress with increased activity of auxiliary breathing muscles, pH <7.25, PaCO_2_ > 45 mm Hg or PaO_2_/FiO_2_<100 mmHg), hemodynamic failure, or arrhythmias and psychiatric symptoms requiring drug intervention.

### 2.4 Statistical analysis

Normally distributed continuous variables were expressed as mean (standard deviation, SD), and continuous variables with skewed distribution were expressed as median (interquartile range, IQR). The Shapiro-Wilk test was used for normality testing. The Student’s t-test was used to compare two groups of equal variances. For non-normal distribution parameters, the Mann–Whitney *U* test was used for statistical comparisons between two groups. To determine the prediction value of the best pendelluft volume for predicting failure weaning, we calculated the area under the receiving operating characteristic curve (ROC). Spearman correlation analysis was used to analyze the correlation between Edi parameters and pendelluft. Statistical analysis was performed using IBM SPSS Statistics version 20 (IBM Corp, Armonk, NY, US), with a *p*-value less than 0.05 considering significant.

## 3 Results

### 3.1 Patients’ characteristics

A total of 60 patients were included, including 50 in the success group and 10 in the failure group. The weaning failure rate was 16.7%. The participants included 40 males and 20 females with a median age of 69.23 (57–85). There were no significant differences in gender, predicting body weight, age, and Acute Physiology and Chronic Health Evaluation (APACHE II) score between the two groups. Among the causes of mechanical ventilation, acute lung disease was most common (43%), followed by cardiac macrovascular disease (25%) and septic shock (10%). The length of stay in ICU (10.8 ± 7.1 days vs. 19.4 ± 7.8 days, *p* <0.001) and 28-day ventilator-free days in the failure group were significantly longer than those of the success group [23(20–25) vs. 0.5(0–6), *p* <0.001]. The 28-day mortality was 70% in the failure group and 4% in the success group. The characteristics of the patients were shown in Table 1.

**TABLE 1:** Clinical characteristics and outcomes of patients.

Characteristics	Success group (n = 50)	Failure group (n = 10)	*p* valve
Male, n (%)	34(68)	6(60)	0.62
Age, years (median, IQR)	71(56.8–80.5)	75.5(62.3–81)	0.73
PBW (median, IQR)	59.6(50.6–64.2)	54.6 (50.1–64.1)	0.78
APACHE II, (mean, SD)	20.2 ± 7.1	19.1 ± 4.0	0.66
Causes of MV, n (%)			
Acute lung disease	21(42)	5(50)	0.64
Cardiac disease	14(28)	1(10)	0.23
Septic shock	5(10)	1(10)	1
Trauma	4(8)	1(10)	0.83
Chronic lung disease	4(8)	2(20)	0.25
Gastrointestinal surgery	2(4)	1(10)	0.43
ICU stay, days	10.8 ± 7.13	19.4 ± 7.8	0.001
28-day mortality, n (%)	2(4)	7(70)	<0.001
28-day ventilator-free days, days (median, IQR)	23(20–25)	0.5(0–6)	<0.001

Abbreviations: APACHE, acute physiology and chronic health evaluation; ICU, intensive care unit; PBW, predicting body weight; MV, mechanical ventilation; IQR, interquartile range.

### 3.2 Respiratory parameters during the baseline and SBT

Both respiration rate and RSBI were significantly differences between the success group and the failure group during SBT. Electrical activity of the diaphragm was gradually enhanced with the increase of spontaneous breathing during SBT. Edimax and ΔEdi were increased faster in the failure group than those in the success group, and Edimax and ΔEdi were significantly higher in the failure group than those in the success group (*p* <0.05) (Table 2).

**TABLE 2:** Respiratory parameters during the baseline and spontaneous breathing trial for all patients.

		All(n = 60)	Success group(n = 50)	Failure group(n = 10)	*p*-value
Vt	T0	494.12(424.01–579.71)	519.68(454.32–615.64)	414.69(360.11–484.74)	0.012
	T1	460.21 ± 126.46	473.75 ± 129.03	392.49 ± 89.87	0.063
	T2	428.69 ± 131.50	439.90 ± 133.40	372.60 ± 110.85	0.141
	T3	437.75 ± 139.05	448.98 ± 140.13	381.6 ± 125.02	0.164
RR	T0	13.74(12.01–16.88)	13.01(12–16.54)	16.72(16.11–18.95)	0.001
	T1	15.56(13.02–18.6)	14.93(12.80–18.22)	18.61(16.07–20.7)	0.015
	T2	17.36 ± 5.03	16.70 ± 4.99	20.67 ± 3.93	0.017
	T3	16.76(13.88–20.15)	16.27(13.30–19.88)	20.77(17.99–22.94)	0.028
RSBI	T0	30.33(20.92–37.95)	26.94(19.52–34.67)	42.32(35.88–53.63)	0
	T1	35.52(26.78–46.40)	32.87(24.14–45.18)	50.05(38.55–63.52)	0.007
	T2	39.97(29.01–59.02)	37.71(27.18–57.37)	58.45(46.23–63.78)	0.036
	T3	41.12(27.15–58.59)	39.19(23.99–55.45)	57.66(46.63–69.06)	0.026
Edimax	T0	4.43(2.22–6.19)	3.92(2.09–5.86)	8.04(3.10–11.11)	0.033
	T1	5.96(2.85–10.21)	4.835(2.77–8.53)	11.5(9.52–17.46)	0
	T2	6.2(2.93–11.26)	4.725(2.78–9.63)	11.37(9.18–13.01)	0.003
	T3	6.65(3.25–11.9)	5.09(3.16–10.42)	12.02(9.18–16.61)	0.003
ΔEdi	T0	3.62(1.76–5.81)	3.23(1.54–5.39)	7.23(2.77–10.44)	0.028
	T1	4.99(2.66–9.30)	4.49(2.27–7.68)	11.13(8.47–16.22)	0
	T2	5.69(2.73–10.29)	4.22(2.26–8.30)	10.64(8.74–12.24)	0.002
	T3	5.46(2.89–10.65)	4.545(2.64–9.82)	11.26(8.82–16.1825)	0.002
Ph	T0	7.46 ± 0.06	7.46 ± 0.06	7.46 ± 0.04	0.887
	T3	7.43 ± 0.06	7.43 ± 0.05	7.43 ± 0.07	0.962
PaCO2	T0	37.35(32.85–41.8)	37.35(32.73–41.6)	38.8(33.03–42.43)	0.513
	T3	39.05(35.725–42.6)	39.15(35.88–42.6)	37.35(34.55–47.35)	0.758
PaO2	T0	101.15(81.2–119.75)	104(86.6–121.5)	76.7(66.6–115.25)	0.04
	T3	99.85(87.175–120)	101(89.58–121.25)	92.4(70.5–117)	0.284

Abbreviations: Vt, Tidal volume; RR, Respiratory rate; RSBI, rapid shallow breathing index; Edi, electrical activity of the diaphragm; T0, 10 min baseline; T1, SBT_10 min; T2, SBT_20 min; T3, SBT_30 min.

### 3.3 Pendelluft volume amplitude during SBT

**FIGURE 3:**
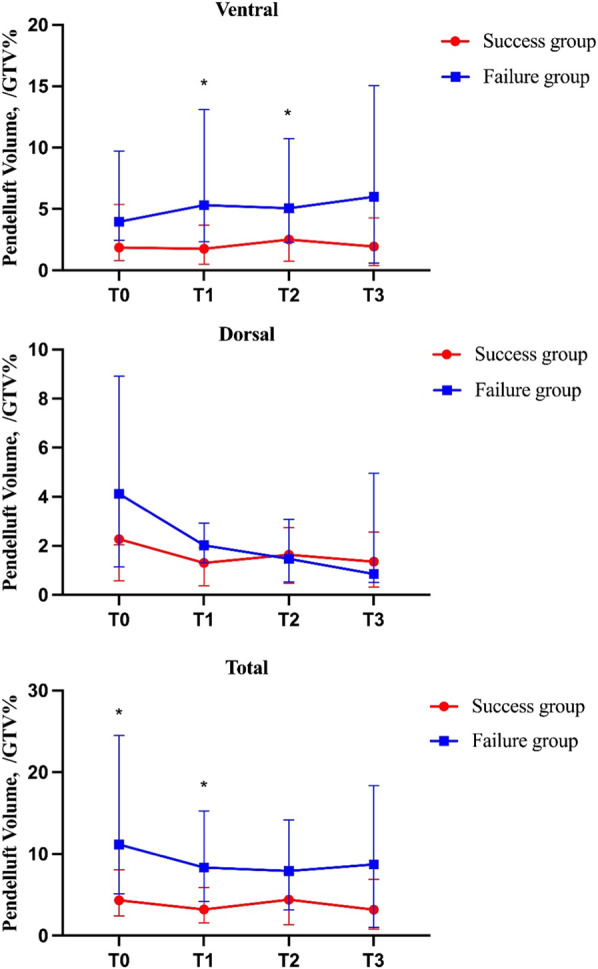
Pendelluft amplitude in each region measured by electrical impedance tomography (EIT) during SBT. EIT imaging was divided into two regions of interest: dorsal and ventral, Total = dorsal + ventral. Pendelluft volume was recorded every 10 min including 10 min baseline(T0), SBT_10 min(T1), SBT_20 min(T2), SBT_30min(T3) with EIT./GTV%, pendelluft amplitude to was tidal variation ratio in percent. The curves in the figure represent the median and upper and lower quartiles.

Compared with the success group, the pendelluft in regions of interest was more obvious in the failure group during SBT. The pendelluft was mainly concentrated in the early stage of the SBT. At T1, total pendelluft volume was significantly higher in the failure group than that in the success group [median 8.34% GTV (interquartile range [IQR] 4.22–15.27) versus median 3.2% GTV (IQR 1.56–5.88), *p* = 0.01]. Ventral pendelluft was more prominent in the failure group than that in the success group at T1 [median 5.3% GTV (IQR 2.33–13.09) versus median 1.75% GTV (IQR 0.50–3.67), *p* = 0.01] and T2[median 5.05% GTV (IQR 2.28–10.72) versus median 2.51% GTV (IQR 0.74–4.70), *p* = 0.032] (Figure 3). Dorsal pendelluft in the failure group showed a downward trend during SBT, which was higher than that in the success group in the first 10 min of SBT (T1), but there was no significant difference between the two groups (*p* >0.05). Through correlation analysis, it was found that the pendelluft was positively correlated with Edi, in which Edimax was positively correlated with the total pendelluft (r = 0.51, *p* <0.05), and Δ Edi was positively correlated with total pendelluft (r = 0.52, *p* <0.05).

### 3.4 The value of pendelluft volume in weaning prediction

**FIGURE 4:**
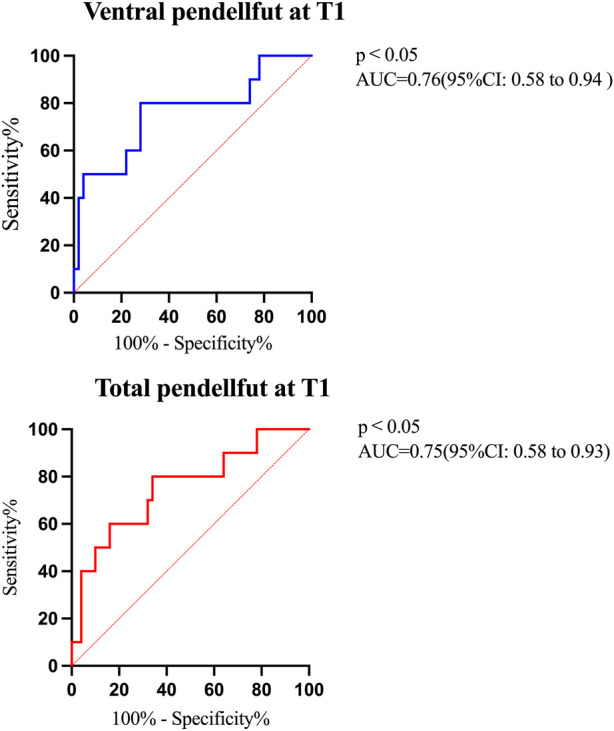
Areas under the ROC curves for the pendelluft volume during SBT. Blue line denote ROC for ventral pendelluft at T1 to failure weaning (AUC = 0.76, 95% CI: 0.58–0.94, cutoff = 3% of global tidal impedance variation); Red line was ROC for total pendelluft at T1 to predict failure weaning (AUC = 0.75, 95% CI: 0.58–0.93, cutoff = 5% of global tidal impedance variation).

ROC curve analysis showed that when the ventral pendelluft volume at T1 was >3% GTV, the area under the curve was 0.76 (95% CI: 0.58–0.94), and the sensitivity and specificity of predicting weaning failure were 72.0% and 80.0%, respectively. Assuming the cutoff value for the total pendelluft volume of being more than 5% GTV at T1, the sensitivity was 66.0% and the specificity was 80.0% (AUC = 0.75, 95% CI: 0.58–0.93) (Figure 4).

## 4 Discussion

Our study found that the pendelluft at the early stage of SBT was more likely to occur in patients with weaning failure, which may be related to diaphragm dysfunction. The monitoring of ventral pendelluft at the early stage of SBT could predict the outcome of weaning. Weaning remains a challenge for clinicians. The rate of weaning failure in this study was 16.7%, which was similar to the previous report (Burns et al., 2021). However, in fact, the rate of weaning failure was higher for patients with difficult-to-wean (Schonhofer et al., 2020).

SBT is a commonly used test to assess whether a patient can be able to wean from the ventilator. Some evaluation indicators, such as RSBI, are the most widely used predictors to predict the outcome, but remain controversial (Trivedi et al., 2021) in predicting weaning for ICU patients. In our study, RSBI of both groups was less than 105. So it is difficult to distinguish the outcome of the weaning by RSBI>105.

The prediction parameters currently used were difficult to show the distribution of lung gas during SBT which might play a key role in the weaning process (Longhini et al., 2019). Bickenbach et al. (2017) proposed that tidal impedance variation (TIV) and the changes in the values of end-expiratory lung impedance (ΔEELI) were significantly decreased, and the global inhomogeneity (GI) was significantly increased during the T-piece trial.

Longhini et al. (2019) used the CPAP to perform SBT. Compared with those who had successful SBT, the patients who failed SBT had a greater decrease in ∆ EELI and a greater pulmonary ventilation inhomogeneity, suggesting that weaning failure was characterized by decreased lung heterogeneity and reexpansion. As we know, spontaneous breathing might improve gas exchange and lung ventilation. However, if the patient was not ready and withdraw mechanical assistance, it would lead to excessive spontaneous breathing, which could also be potentially harmful and lead to lung injury (Carteaux et al., 2021; Chi et al., 2022).

Yoshida et al. (2013) found pendelluft phenomenon with shift of air from non-dependent to dependent lung regions during spontaneous respiration through EIT technology*.* The local negative pleural pressure generated by diaphragmatic contraction is not uniformly transmitted but concentrated in the injury lung. The high level of support during SBT did not show significant differences in pendelluft, but with the decrease of support pressure, the volume of pendelluft doubled, and the gas moved from the ventral to the dorsal side (Coppadoro et al., 2020). In our study, ventral pendelluft was more obvious in the patients with weaning failure, which indicated that alveolar overdistension in the ventral and collapse in the dorsal.

Our study found that Edimax and ΔEdi were significantly higher in patients in the failure group than those in the success group, and Edimax an d ΔEdi in the failure group increased significantly in the early stage of SBT(T1) (Table 2). Barwing et al. (2013) confirmed that a sharp increase in electrical activity of the diaphragm was observed 5 min after disconnection from the ventilator. We jointly monitored the change trends of pendelluft and electrical activity of the diaphragm during SBT for the first time, and found that the pendelluft in early SBT was positively correlated with the electrical signal change of diaphragm, suggesting that pendelluft might be related to diaphragm dysfunction. This provides a research idea for future study of the physiological function of diaphragm in the process of weaning.

### 4.1 Study limitations

There are a few limitations to our study. Firstly, it was a single center study with small sample size. Secondly, our study did not include different modes of SBT, and did not set different pressures in the low level of pressure support during SBT. This could ignore the pulmonary compliance and etiology of respiratory failure in different patients (Burns et al., 2017). Thirdly, this study only included patients with initial SBT, which might result in inaccurate assessment of difficult-to-wean patients. Fourthly, the correlation between diaphragm function and pendelluft needed more parameters of diaphragm function, such as ultrasound (Kilaru, Panebianco, and Baston, 2021). Finally, throughout the weaning process, ventral pendelluft and total pendelluft in the failure group were higher than those in the success group, but there was no significant statistical difference at T3, which could be related to the fact that a small sample size might cause low statistical power in our current study. Further large controlled studies will be conducted to validate these findings. Furthermore, we did not perform a subgroup analysis of pendelluft among patients with difficult weaning, which will be another research focus in our future work.

In conclusion, pendelluft was more obvious in the patients who failed to wean from the ventilator, which was one of the factors that cause weaning failure. Quantifying the pendelluft volume has certain predictive value for weaning failure.

## Data Availability

The original contributions presented in the study are included in the article/supplementary material, further inquiries can be directed to the corresponding authors.
